# Kynurenine pathway metabolites are associated with gray matter volume in subjects with schizophrenia

**DOI:** 10.3389/fpsyt.2022.941479

**Published:** 2022-08-09

**Authors:** Sumiao Zhou, Yuanyuan Huang, Qijie Kuang, Su Yan, Hehua Li, Kai Wu, Fengchun Wu, Xingbing Huang

**Affiliations:** ^1^The Affiliated Brain Hospital of Guangzhou Medical University, Guangzhou, China; ^2^School of Biomedical Sciences and Engineering, South China University of Technology, Guangzhou, China; ^3^Guangdong Engineering Technology Research Center for Translational Medicine of Mental Disorders, Guangzhou, China

**Keywords:** kynurenine, kynurenic acid, gray matter volume, schizophrenia, kynurenine metabolism

## Abstract

**Background:**

There has been growing evidence of the existence of abnormalities in the kynurenine pathway (KP) and structural gray matter volume (GMV) in schizophrenia (SCZ). Numerous studies have suggested that abnormal kynurenine metabolism (KM) in the brain is clearly associated with the pathogenesis of schizophrenia and may be one of the pathological mechanisms of SCZ. In this pilot study, we investigated whether there was a correlation between KP and GMV in schizophrenia patients.

**Methods:**

The plasma levels of KM were measured in 41 patients who met the Structured Clinical Interview of the Diagnostic IV criteria for schizophrenia and 60 healthy controls by using liquid chromatography-tandem mass spectrometry, and cortical thickness (as measured *via* magnetic resonance imaging) was obtained.

**Results:**

Our study showed no statistically significant differences in the concentrations of kynurenine (KYN), tryptophan (TRP), and KYNA/TRP (all *p* > 0.05), but kynurenic acid (KYNA) and the KYNA/KYN ratio were significantly higher in the schizophrenia subjects than in the healthy controls (*F* = 4.750, *p* = 0.032; *F* = 6.153, *p* = 0.015, respectively) after controlling for age and sex. Spearman's tests showed that KYN concentrations in SCZ patients were negatively correlated with GMV in the left front cingulate belt (*r* = −0.325, *p* = 0.046) and that KYN/TRP was negatively correlated with GMV in the left island (*r* = −0.396, *p* = 0.014) and right island (*r* = −0.385, *p* = 0.017).

**Conclusion:**

Our findings appear to provide new insights into the predisposition of an imbalance in the relative metabolism of KYN/TRP and KYN to GMV in schizophrenia.

## Introduction

Schizophrenia (SCZ), a psychiatric disorder characterized by psychotic symptoms and results in social and occupational declines, remains an aetiological and therapeutic challenge (1). The upregulation of the immune response has been reported to be possibly involved in the pathogenesis of SCZ (2, 3). The Kynurenine pathway (KP) is regulated by the immune system and the levels of KYNA are well regulated under physiological conditions but may be altered as part of the activated immune response (4–7). There has been growing evidence of the existence of abnormalities in the KP in SCZ.

A meta-analysis that included 4,217 participants and 42 studies examined the KYN pathway in schizophrenia, and the results demonstrated higher KYN levels in the cerebrospinal fluid and lower plasma KYN levels (8). The kynurenic acid (KYNA)-to-KYN ratio is decreased in major depressive disorder (MDD) and SCZ, and the KYN-to-tryptophan (TRP) ratio is increased in MDD and SCZ in blood (plasma or serum) (9). In contrast to the replicable findings of the elevation of KYNA in the central nervous system in individuals with schizophrenia spectrum disorder, plasma levels of KYNA were significantly lower in SCZ patients than in controls (6). All of the above mentioned studies support the existence of abnormal KM in the brains of SCZ patients, mainly in regards to the upregulation of KYN and KYNA levels, thus suggesting that abnormal KM in the brain is clearly associated with the pathogenesis of SCZ and may be one of the pathological mechanisms of SCZ.

Kynurenine pathway (KP) is the major metabolic pathway for tryptophan (TRP) (10, 11). Within central nervous system, TRP is degraded to KYN *via* indoleamine-2, 3-dioxygenase and tryptophan-2, 3-dioxygenase (11–14). The decrease in KYN may contribute to the development of chronic mild inflammation (8, 15). It has also been reported that inflammatory factors can also directly or indirectly affect the metabolic pathways of KYN in patients with SCZ under the induction of inflammatory states (16, 17). It can be mainly metabolized into KYNA *via* kynurenine 66 aminotransferase (KAT) in neurons, and it can be metabolized into 3-hydroxykynurenine (3-OHK) *via* kynurenine 3-monooxygenase, which is further catabolized to quinolinic acid (QA) finally (18–21). KYNA, which is a neuroprotective tryptophan catabolite, is an antagonist of N-methyl-D-aspartate receptors (NMDARs) and is considered to be protective against excitotoxic and apoptotic effects (16, 22–24). However, increased KYNA in the brain may lead to excessive NMDAR blockade, which is a known trigger of psychotic symptoms in SCZ (22, 25). QA is a neurotoxic catabolite that infiltrates macrophages, activates N-methyl-D-aspartate (NMDA) receptors, and exerts neurotoxic effects as a glutamate N-methyl-D-aspartate (NMDA) receptor agonist (17). An increase in QA and a decrease in KYNA would result in NMDAR activation to further release reactive oxygen species under mitochondrial oxidative stress, thus leading to cell damage (8, 26, 27).

The reduction and loss of GMA is a common feature of both early and chronic phases of SCZ (28–30). Relative to healthy controls, patients with SCZ had reduced gray white matter in the bilateral precentral gyrus, right medial frontal cortex, right visual cortex, right occipital pole, right thalamus, bilateral amygdala, and bilateral cerebellum, regardless of substance use history (7, 31, 32). Furthermore, SCZ demonstrated some evidence of GMV loss in cortical areas; however, notable locations included limbic structures such as the hippocampus, thalamus and striatum, and cerebellum in a regional first-episode psychosis MRI study (33). In general, reduced and thinner cortical thickness is more common in patients with SCZ (34).

It is of interest that Kyn/3 HK was also significantly associated with the total volume of the amygdala in the bipolar disorder group (17). The results indicate the possibility that bipolar disorder-associated abnormalities in KM may impact the structure of the hippocampus and amygdala. Based on the previously discussed literature, we hypothesized that the putative neuroprotective index kynurenine pathway metabolites would significantly correlate with GMV in patients with SCZ.

## Methods

### Subjects

41 SCZ patients (all outpatients) from the Affiliated Brain Hospital of Guangzhou Medical University were recruited during the period from January 2017 to December 2018. Patients who (1) met the Structured Clinical Interview of the Diagnostic IV criteria for schizophrenia, (2) aged 18–50 years, (3) had at least 6 years of education, and (4) were currently in the acute phase of SCZ with a total PANSS score ≥ 60 were enrolled.

The exclusion criteria were as follows: (1) severe physical diseases, such as severe heart disease and thyroid disease; (2) previous histories of brain trauma, mental retardation, and epilepsy, among other histories; (3) those patients who had used electroconvulsive therapy 6 months prior to enrolment; and (4) those patients with contraindications to MRI scanning.

Healthy controls were included from the Guangzhou community during the same time period, from which 60 healthy subjects matched for age, sex, and education level were screened as the healthy control (HC) group for this study.

The HCs met the same exclusion criteria except that they had (1) good physical health with no history of specific physical illness and (2) no family history of psychiatric disorders.

### Plasma KM measure

All of the blood samples were obtained from each subject in the morning on an empty stomach and completed the MRI scan within 48 h. Peripheral blood specimens were centrifuged and separated into serum, plasma, and blood cells, after which they were immediately stored in an ultralow temperature refrigerator at−80°C to be measured. The plasma tryptophan, kynurenine, and kynurenic acid levels (as assessed by high-performance liquid chromatography-tandem mass spectrometry) were blinded to diagnosis in the central laboratory of the Brain Hospital of Guangzhou Medical University.

A 1,200 high performance liquid chromatograph and a 6,410 triple quadrupole tandem mass spectrometer from Agilent (USA) were used for the determination of the kynurenine metabolites. The chromatographic conditions: Agilent Eclipse XDB-C18 column (4.6–150 mm, 5um) as stationary phase, TRP-KYNA mobile phase of methanol-water (45:55, containing 0.005 mol/l ammonium formate), KYNA mobile phase of methanol-water (35:65, containing 0.005 mol/l ammonium formate) The flow rate was 0.5 ml/min, and the column temperature was 35°C. Mass spectrometry conditions: TRP (m/z 205.1 → 199.1), KYN (m/z 209.1 → 146.1) and deuterated internal standards KYN-^13^C_4_, 15N (m/z 214.1 → 149.1), KYNA (m/z 190.1 → 144.1) and deuterated internal standard KYNA-d_5_ (m/z 195.1 → 149.1). Linearity range: TRP: 1–50ug/ml; KYN: 0.1–5ug/ml; KYNA: 1–60ug/ml.

### Clinical assessment

Psychiatric symptoms and severity of schizophrenia were primarily assessed by two trained attending psychiatrists (Consensus Kappa ≥ 90%) by using the Positive and Negative Syndrome Scale (PANSS).

### Structural MRI data preprocessing

All 3D T1 structural MRI data were processed on the SPM8 (Wellcome Department of Imaging Neuroscience Group, UK; http://www.fil.ion.ucl.ac.uk/spm) based VBM (voxel-based morphometry) (http://dbm.neuro.uni-jena.de/vbm.html) toolkit for preprocessing. Structural MRI data preprocessing includes the following steps.

1. Adjustment of origin: the origin of T1 structural MRI data was manually adjusted to the anterior joint position or nearby, which facilitated a better alignment of the structural image data to the MNI standard space.
2. Spatial normalization: we applied the high-dimensional DARTEL method to normalize the T1 structural image space to the MNI standard space.
3. Segmentation: after spatial normalization, the image was segmented into gray matter, white matter, and cerebrospinal fluid.
4. Modulation: in the spatial normalization process, the segmented gray matter images were modulated to eliminate the volume variation due to the individual differences in the subject's brain.
5. Smoothing: for the next step of the statistical analysis, a Gaussian kernel with full-width at half maximum was used to smooth the segmented and modulated data.

### Statistical analysis

All of the statistical tests that were conducted were two-tailed. Deviations from normality were tested by using the Kolmogorov–Smirnov test, and non-normally distributed variables were log normalized. The data, such as age, education, and course of the disease, conformed to a normal distribution and were tested by using independent sample *t*-tests for both groups. Categorical variables were compared between the SCZ and HC groups by using chi-square tests. An analysis of variance was used to test for differences in TRP, KYN, KYNA, KYN/TRP, and KYNA/KYN between the SCH and HC groups after controlling for age and sex. For kynurenine metabolites that were statistically correlated with GMV, we subsequently conducted partial correlation to control for potential confounders (sex, age, and intracranial volumes) in schizophrenia.

## Results

### Demographic and clinical characteristics of the SCZ and HC groups

A total of 41 patients with SCZ were enrolled in this study, of whom 22 (53.7%) patients were male and 19 (46.3%) patients were female. There were 60 HC subjects, including 30 (50%) males and 30 (50%) females. No significant differences were found between SCZ patients and HCs in terms of age, sex, or education level (*p* > 0.05; Table 1).

**Table 1:** Demographic and clinical characteristics of SCZ patients and HCs.

**Variables**	**SCH**	**HC**	**t/χ^2^**	***p-*value**
	**(*n* = 41)**	**(*n* = 60)**		
Age	27.8 ± 7.3	30.2 ± 11.5	1.286^a^	0.238
Gender (male/female)	22/19	30/30	0.840^b^	0.718
education (years)	11.2 ± 2.9	10.4 ± 1.81	−1.50^a^	0.232
Course of disease (months)	44.3 ± 56.3	NA	NA	NA
**Antipsychotics (** ***n*** **%)**			
Olanzapine	10%			
Risperidone	10%	NA	NA	NA
Amisulpride	5%			
Clozapine	3%			
P subscore	24.4 ± 3.7	NA	NA	NA
N subscore	21.6 ± 6.4	NA	NA	NA
G subscore	41.9 ± 6.9	NA	NA	NA

a Independent samples t-test;

b Chi-square test,. P, positive symptoms; N, negative symptoms; G, general psychopathology syndrome; PANSS, Positive and Negative Syndrome Scale; NA, Not applicable.

### Plasma kynurenine metabolite levels in the SCZ and HC groups

After controlling for age and sex, no statistically significant differences in the concentrations of KYN, TRP, and KYNA/TRP were found between the SCZ subjects and the HCs. However, Table 2 shows that the KYNA level and KYNA/KYN ratio were significantly higher in the SCZ subjects than in the HCs (*F* = 4.750, *p* = 0.032; *F* = 6.153, *p* = 0.015; respectively) after controlling for age and sex.

**Table 2:** Comparison of KM between patients with SCZ and HCs.

**Variables**	**SCZ (*n* = 41)**	**HC (*n* = 60)**	**F**	***p-*value**	**Cohen's d**
TRP (ng/mL)	8376.01 ± 2340.30	8379.69 ± 1228.73	0.005	0.943	−0.002
KYN (ng/mL)	302.58 ± 87.77	312.68 ± 98.84	0.115	0.736	−0.107
KYNA (ng/mL)	6.85 ± 2.67	5.87 ± 1.84	4.750	**0.032**	0.440
KYN/TRP	0.04 ± 0.11	0.04 ± 0.02	0.014	0.905	−0.026
KYNA/KYN	0.03 ± 0.01	0.02 ± 0.01	6.153	**0.015**	0.508

SCZ, Schizophrenia; HC, healthy controls; TRP, tryptophan; KYN, kynurenine; KYNA, kynurenic acid. Bolded values are p < 0.05.

### Comparison of GMV between the SCZ and HC groups

Table 3 shows that the comparison of GMV brain regions in the SCZ patient groups and the HC group was analyzed by using covariance, and the gray matter volumes in the left anterior cingulate gyrus, left insula, right insula, right middle cingulate gyrus, right cingulate gyrus, and left inferior temporal gyrus were lower in the SCZ group than in the HC group. After the analysis of covariance and correction for the false discovery rate, there was still a significant difference between the schizophrenia subjects and the healthy controls (all *p*-values < 0.01).

**Table 3:** The different brain areas of GMV between SCZ and HC.

**Brain area**	**SCZ (*n* = 41)**	**HC (*n* = 60)**	**F**	***p-*value**	**Cohen's d**
Front cingulate belt (Left)	0.58 ± 0.06	0.64 ± 0.06	42.408	**<0.001**	−1.000
Island (Left)	0.60 ± 0.06	0.66 ± 0.07	45.411	**<0.001**	−0.906
Island (Right)	0.49 ± 0.06	0.55 ± 0.08	32.795	**<0.001**	−0.825
Middle cingulate gyrus (Right)	0.55 ± 0.06	0.62 ± 0.06	41.356	**<0.001**	−1.167
Fusiform gyrus (Right)	0.55 ± 0.06	0.63 ± 0.10	33.352	**<0.001**	−0.926
Inferior temporal gyrus (Left)	0.64 ± 0.07	0.71 ± 0.06	33.385	**<0.001**	−1.091

SCZ, schizophrenia; HC, healthy controls. Bolded values are p < 0.05.

### The correlation between KM and GMV in SCZ

As shown in Table 4, the biased correlation results showed that KYN concentrations in SCZ patients were negatively correlated with GMV in the left anterior cingulate gyrus (*r* = −0.325, *p* = 0.046) and that KYN/TRP was negatively correlated with GMV in the left insula (*r* = −0.396, *p* = 0.014) and right insula (*r* = −0.385, *p* = 0.017).

**Table 4:** The Correlation between KM and GMV in SCZ.

**Variables**		**TRP**	**KYN**	**KYNA**	**KYN/TRP**	**KYNA/KYN**
Front cingulate belt (Left)	*r*	−0.093	−0.325	−0.041	−0.201	0.177
	*p*	0.579	**0.046**	0.806	0.227	0.289
Island (Left)	*r*	0.104	−0.253	−0.133	−0.396	0.016
	*p*	0.536	0.126	0.426	**0.014**	0.924
Island (Right)	*r*	0.128	−0.218	−0.046	−0.385	0.105
	*p*	0.444	0.189	0.783	**0.017**	0.529
Middle cingulate gyrus (Right)	*r*	0.005	−0.161	0.03	−0.163	0.091
	*p*	0.976	0.334	0.859	0.328	0.587
Fusiform gyrus (Right)	*r*	−0.138	−0.109	0.16	0.06	0.252
	*p*	0.408	0.514	0.337	0.721	0.128

SCZ, schizophrenia; HC, healthy controls; TRP, tryptophan; KYN, kynurenine; KYNA, kynurenic acid. Bolded values are p < 0.05.

## Discussion

In this study, we observed an increase in the neuroprotective index, KYNA, and KYNA/KYN in the schizophrenia group vs. the healthy controls; however, TRP and KYN were not altered. The main finding of this study was that within the schizophrenia group, KYN concentrations were negatively correlated with GMV in the left anterior cingulate gyrus, and KYN/TRP was negatively correlated with GMV volume in the left insula and right insula.

Consistent with our hypothesis and previous studies, we observed abnormalities in kynurenine pathway metabolism in patients with schizophrenia, and KYNA concentrations and the KYNA/KYN ratio were higher in schizophrenia patients (3, 26). This finding further validates a disturbance in the relative balance of activation of the KYNA pathway in schizophrenia (35). It is hypothesized that the activity of the rate-limiting enzyme related to KP is abnormal in schizophrenic patients, and it appears that these two substances with opposite activities of action may be out of balance and tend to favor the production of KYNA (36). The explanation for the increased KYNA/KYN ratio seems unambiguous because the high levels of kynurenine-3-monooxygenase and all other cerebral kynurenine aminotransferases allow for a proportional increase in KYNA formation when KYN levels rise (37). Thus, there is evidence supporting the hypothesis that KP metabolites can be used as peripheral markers of brain dysfunction in schizophrenia (26).

We found extensive GMV loss in several brain regions, including the left anterior cingulate gyrus, left insula, right insula, right middle cingulate gyrus, right cingulate gyrus, and left inferior temporal gyrus, in schizophrenia patients, which is consistent with a greater number of previous studies (4, 38–40). It has been reported that individuals with schizophrenia showed reduced GMV in previously identified areas of the prefrontal cortex, frontal lobes, cerebellum, and thalamus (22, 41). Furthermore, McEwen et al. (42) noted that total (left and right) prefrontal GMV was significantly reduced in first-episode schizophrenia. The reduction in GMV is a common feature of both the early and chronic phases of schizophrenia.

In the present study, we extended the analysis to include KYNA and GMA. This is the first study to report on the correlation between KM and GMV in patients with schizophrenia. Our findings showed that the left anterior cingulate gyrus, left insula, and right insula are strongly associated with KP, that KYN concentration was negatively correlated with GMV in the left anterior cingulate gyrus, and that KYN/TRP was negatively correlated with GMV in the left insula and right insula. Intriguingly, this suggests that the activation of the KP with increased KM activity is associated with decreased GMV in certain relevant brain regions; additionally, it is further hypothesized that the activation of KP may lead to an imbalance between neurotoxicity and neuroprotection of the biochemical pathway of KM, thus causing significant changes in GMV in these brain regions (17, 43).

Similar to our study, Chiappelli et al. (44) showed that the KYN/TRY ratio was inversely correlated with frontal white matter glutamate in a study that included 37 SCZ and 38 HCs to detect levels of total tryptophan and its metabolite kynurenine. In addition, a study in which 63 bipolar depressed patients and 48 HCs completed structural MRI scans and blood samples and were analyzed for canine urinary quinoline metabolites also found that KynA/3HK was positively associated with hippocampal volume in the BD group (17). These results provide preliminary evidence that plasma levels of KYN and KYN/TRP can provide a readily measurable peripheral indicator of the degree of GMA brain volume loss in schizophrenia patients.

This study had a number of limitations. For example, this was a cross-sectional study with a small sample. A small percentage of patients were on antipsychotic medication, and we failed to limit the use of the medication. In addition, this study only tested the levels of KM in the blood and did not test the levels of KM in the cerebrospinal fluid. Therefore, in future studies, there is a need to further expand the sample size to include first-ever patients who have never taken the drug, in order to restrict the enrolment sample more strictly, to test the levels of kynurenine metabolites in both the cerebrospinal fluid and blood of patients, and to perform dynamic comparative observations and evaluations before and after treatment. In this way, more in-depth analytical studies can be conducted to further validate our proposed hypothesis.

## Conclusion

Our findings revealed that an altered biomarker of interaction with cortical structure, and KP is related to GMV integrity in schizophrenia. All our findings provided new insights to into the predisposition of an imbalance in the relative metabolism of KYN/TRP and KYN to GMV in schizophrenia. Although the data in this study are not sufficient to pinpoint the underlying mechanism of this relationship.

## Data availability statement

The data presented in the study are deposited in the figshare repository, https://doi.org/10.6084/m9.figshare.20391297.

## Ethics statement

The studies involving human participants were reviewed and approved by the Ethics Committee of The Affiliated Brain Hospital of Guangzhou Medical University. Written informed consent to participate in this study was provided by the participants' legal guardian/next of kin.

## Author contributions

XH and FW revised the manuscript. SZ were responsible for interpreting the data and drafting the manuscript. YH, QK, and SY participated in the patient recruitment and analysis of all of the data. HL and KW assisted with the primary study design. All of the authors read and approved the final manuscript.

## Funding

This research was supported by the National Key Research and Development Program of China (Grant No. 2021YFC2009403), the Scientific Research Project of Traditional Chinese Medicine of Guangdong (Grant No. 20192070), the Traditional Chinese Medicine Bureau of Guangdong Province (Grant No. 20211305), and the Science and Technology Plan Project of Guangdong Province (Grant No. 2019B030316001).

## Conflict of interest

The authors declare that the research was conducted in the absence of any commercial or financial relationships that could be construed as a potential conflict of interest.

## Publisher's note

All claims expressed in this article are solely those of the authors and do not necessarily represent those of their affiliated organizations, or those of the publisher, the editors and the reviewers. Any product that may be evaluated in this article, or claim that may be made by its manufacturer, is not guaranteed or endorsed by the publisher.
